# Complexities of Adherence and Post-Cancer Lymphedema Management

**DOI:** 10.3390/jpm5040370

**Published:** 2015-11-16

**Authors:** Pamela L. Ostby, Jane M. Armer

**Affiliations:** 1 Sinclair School of Nursing, University of Missouri, Columbia, MO 65211, USA; E-Mail: armer@missouri.edu; 2 Lymphedema Research Laboratory, Sinclair School of Nursing, University of Missouri, DC 116.05, Suite 408, Mizzou North Campus, Columbia, MO 65211, USA

**Keywords:** adherence, breast cancer, compliance, lymphedema, management, self-care, symptom distress, treatment burden

## Abstract

Breast cancer survivors are at increased risk for breast cancer-related lymphedema (BCRL), a chronic, debilitating, condition that is progressive and requires lifelong self-management. Up to 40% of 3 million breast cancer survivors in the US will develop BCRL, which has no cure, is irreversible, and requires self-management with regimens that may include multiple components. The complexities of treatment can negatively affect adherence to BCRL self-management which is critical to preventing progressive swelling and infection. The aim of this review of contemporary literature published from 2005–2015 is to examine the complexities of BCRL self-management, to identify adherence-focused studies relevant to BCRL, and to summarize barriers to self-management of BCRL. Six electronic indices were searched from which 120 articles were retrieved; 17 were BCRL-focused; and eight met inclusion criteria. Seventeen of 120 articles identified barriers to self-management of BCRL such as complexities of treatment regimens, symptom burden, balance of time for treatment and life demands, and lack of education and support; however, only eight studies included outcome measures of adherence to BCRL treatment regimens with a subsequent improvement in reduced limb volumes and/or perceptions of self-efficacy and self-regulation. A major limitation is the few number of rigorously developed outcome measures of BCRL adherence. In addition, randomized studies are needed with larger sample sizes to establish adequate levels of evidence for establishing best practice standards for improving adherence to BCRL self-management treatment regimens.

## 1. Introduction

Of three million breast cancer survivors in the United States [1], up to 40% will develop breast cancer-related lymphedema (BCRL), a chronic, debilitating condition with a variety of causes that restrict the flow of lymphatic fluid [2,3]. BCRL occurs more frequently in women who have undergone axillary lymph node dissection, sentinel lymph node biopsy, and/or radiation therapy for treatment of breast cancer [4]. Lymphedema can occur soon after surgery or up to decades postoperatively in survivors. After emergence, it is a life-long condition that requires on-going management of symptoms with daily treatment regimens to prevent progression of serious symptoms that include swelling, loss of sensation, pain, reduced range of motion, and infection [2,4].

There is no standardized patient education for BCRL or universal standardized evidence-based treatment protocol to manage the symptoms of BCRL [5,6]. The goal of BCRL treatment involves moving stagnating lymph fluid to an area where it can drain. Once diagnosed, intensive decongestive therapy is performed by a specialty-trained therapist followed by a prescribed self-management regimen [7]. The ability to care for oneself by self-administration of a prescribed regimen to regulate symptoms and promote well-being encompasses self-management [8]. It is important to facilitate lymph circulation of the affected extremity; therefore, prescribed self-management regimens are often bundled and may number as many as twelve modalities of care, depending on the severity of the lymphedema, leading to significant patient burden [9]. The aim of this contemporary literature review is threefold: (a) to examine the complexities of BCRL self-management; (b) identify the adherence-focused studies relevant to BCRL; and (c) to summarize barriers to self-management of BCRL. Supporting studies relevant to complexities and barriers to treatment are discussed, followed by a review of eight interventional studies that met search criteria using adherence as an outcome measure.

Historically, the term “compliance” referred to a person’s conformity to clinical advice in regard to a prescribed dose, frequency, and time, without any autonomy or independent decision-making on the part of the patient [10,11,12]. The term “adherence” places a focus on patient needs and the relationships between patients and health care providers, and suggests a broader interpretation on understanding the factors such as psychological, behavioral, and personality characteristics that affect a patient’s ability to follow treatment recommendations [11,12]. Although there are many variables that contribute to successful adherence, Vermeire *et al.* reported that the strongest factor that correlates with adherence was patients’ own beliefs based on their knowledge and experiences, as well as those of families and friends [13]. Taking into consideration that both terms have been used interchangeably in the literature, both were used in this search to provide a more inclusive analysis of the complexities in self-care of BCRL. However, for ease in reading, the term, “adherence” is used throughout this review.

Adherence to prescribed self-management regimens is critical to preventing progression of BCRL. Complexities of adherence relevant to self-management of lymphedema symptoms encompass physiological, psychological, and psychosocial factors and have been correlated to low adherence to performing BCRL self-management regimens [7,14,15,16,17]. In addition, the belief in self-efficacy and the ability to control lymphedema are cognitive belief variables that have been correlated with adherence [18]. The complexities of BCRL self-management treatment are a barrier to adherence due to cumbersome and time-consuming treatment regimens. Over the past two decades, average self-management rates have remained sub-optimal, between 40% and 50% [17,19].

There is a need for additional research in the area of adherence to self-management of BCRL; however, this must be preceded by an understanding of the complexities that lead to poor adherence and inability to adhere to self-management regimens.

## 2. Methodology

A search of contemporary literature relative to BCRL treatment and adherence was independently assessed by the first author. Inclusion criteria for this review included: (1) studies pertaining to self-management of BCRL; (2) studies in which an intervention was used to improve symptom management through adherence or compliance to self-management of BCRL; (3) studies that included a specific outcome measure of adherence; (4) studies published in the English language; and (5) studies published between 2005 and 2015. The terms “breast cancer”, “lymphedema”, and “self-management” were applied to six electronic database indices, which included: Academic Search Complete, CINAHL, ERIC, MEDLINE, PsycARTICLES, and PubMed, from which 120 articles were retrieved. Fifty-nine studies unrelated to secondary lymphedema from breast cancer treatment and 39 studies that did not address BCRL self-management were excluded. Of the remaining 22 studies, eight studies met the inclusion criteria in which BCRL adherence was a primary outcome measure. Most studies identified barriers to adherence; however, most lacked information related to valid and reliable instruments used to measure adherence. A systematic literature review is a next step; however, this is not currently possible, given the limited number of studies with tested instruments, thus preventing a statistical comparison of results and preventing the establishment of meaningful levels of evidence in measuring BCRL adherence.

## 3. Complexities of BCRL Adherence

Successful self-management of BCRL is outcome-oriented and focuses on prevention of BCRL progression, decreased limb volume of the affected extremity and/or decreased symptoms, and an increase in range of motion and functionality [20]. Complexities have been defined as a number of intricate variables that are interrelated and which serve as obstacles to successful self-management [21]. The interrelated variables of BCRL management encompass every human dimension, with an emphasis on the patients’ perceptions of the condition and treatment [22].

### 3.1. Psychological Complexities

Few studies have been published about the perceptions of women with BCRL performing daily self-managed treatment regimens [23], although a more recent systematic review commissioned by the American Lymphedema Framework Project (ALFP) reported that lymphedema has a negative impact on individuals who are affected by the condition [15]. There are many factors that contribute to psychological distress which impact patients’ abilities to cope with cancer and treatment for both the disease and subsequent treatment-related comorbidities such as lymphedema [23,24,25]. The National Comprehensive Cancer Network defines distress in cancer as:

“…an unpleasant emotional experience of a psychological (cognitive, behavioral, emotional), social, and/or spiritual nature that may interfere with the ability to cope effectively with cancer, its physical symptoms and its treatment [which] extends along a continuum, ranging from common normal feelings of vulnerability, sadness, and fears to problems that can become disabling, such as depression, anxiety, panic, social isolation, and existential and spiritual crisis”.

[24]

Distress can include symptoms of anxiety and depression and may cause insomnia, lack of appetite, and difficulty concentrating and carrying on normal activities. About one third of all cancer patients experience significant distress, with only 5% of those patients seeking psychological help [24]. In a qualitative study of women breast cancer survivors (N = 13), Rosedale and Fu conducted a secondary analysis of phenomenologic data, examining symptom distress in terms of temporal, situational, and attributive dimensions. Although more prospective studies are needed, common themes suggested a relationship between symptom distress and psychological distress [26].

Psychological, and psychosocial factors have been correlated to low adherence in performing BCRL self-care management regimens [15,16,27]. In a systematic review of the literature between 2004–2011 (N = 23 articles), Fu *et al.* reported negative psychological and psychosocial impact in each of 12 qualitative studies, including negative self-identity, psychological distress, social isolation, public insensitivity, and perceived social abandonment [15]. The magnitude of the relationships between negative psychological and psychosocial factors and BCRL has been documented as a cause of non-adherence to self-management, as well as diminution in quality of life (QOL) [6,17,28,29,30]. In a cross-sectional, mixed-methods QOL study with 128 breast cancer survivors (age-matched within 3 years), of whom 64 had BCRL and 64 did not, Ridner reported scores were significantly lower (*p* < 0.01) on all QOL measures in the lymphedema group, including the functional assessment of cancer therapy (FACT-B) with the FACT-B Plus 4 subscale, the Upper Limb Lymphedema 27 (ULL-27), and the Wesley Clinic Lymphedema Scale (WCLS) [30]. There is a need for health care providers to understand that there is an overlap of psychological and physiological sequelae which has a significant impact on adherence to self-management of chronic conditions, such as BCRL.

### 3.2. Education

Education about lymphedema, treatment, and risk-reduction is an important factor in promoting adherence to self-management; however, knowledge by itself is not a predictor of adherence to risk-reduction behaviors [18,31]. In addition, several studies focus on patient education; however, few incorporate a supportive component. Education by itself is not sufficient to provide the support that patients need. Tsuchiya, Horn, and Ingham reported information provision about lymphedema alone may not lead to improved adherence or help-seeking behaviors [32]. In addition, it was suggested that patients’ perceptions of illness, consistent with Leventhal’s Common Sense Model (CSM), should be considered as necessary in facilitating effective symptom management [32,33]. Leventhal’s CSM of Self-Regulation concepts include: (a) representation of illness; (b) coping; and (c) appraisal [33]. The adaptability of the CSM is one of its main benefits and it can be used with a variety of patients who struggle with chronic disease. Leventhal and colleagues describe five components of illness representations: (1) Identity: the label or name given to the condition and the symptoms that “appear” to go with it; (2) Cause: the patient’s own ideas about the perceived cause of the condition, which may or may not be medically accurate; representations are based on personal experience, opinions of significant others, health care professionals, and media sources which may reflect adverse issues such as stress; (3) Time-line: the predictive belief about how long the condition might last; (4) Consequences: the individual beliefs about the consequences of the condition and how it will affect them both, physically and socially; and (5) Curability/controllability: the beliefs about the degree to which a patient believes they can demonstrate self-efficacy and self-regulation in controlling or managing the condition [34,35]. The appraisal process and choosing effective coping strategies are the basis of improved perceptions of self-efficacy and self-regulation. Leventhal’s CSM of illness representations suggests that patients’ perceptions should be considered in order to facilitate effective symptom management and better adjustment [36,37].

Patients’ perceptions of self-efficacy are also contributing factors to adherence to self-management of BCRL. In a study of women (N = 98) who were scheduled for breast and lymph node surgery, Sherman and Koelmeyer reported that data from questionnaires completed pre- and three months post-surgery demonstrated greater adherence to BCRL risk-reduction behaviors among participants who had greater beliefs of self-efficacy and self-regulatory abilities to control lymphedema. Findings suggest that while inclusion of education is important, it should have a motivational component to facilitate long-term adherence [18]. Another study was conducted by Sherman *et al.* with women who had undergone breast and lymphatic surgery (*N* = 103). The participants completed questionnaires to measure perceived lymphedema risk, beliefs and expectancies, self-regulatory ability, distress, knowledge, and adherence to BCRL risk-reduction behaviors. The women were then given printed information from the American Cancer Society about breast cancer. Cognitive and affective variables were reassessed at six and 12 months post-baseline (*n* = 62). The findings of the study reinforced the importance of education about lymphedema risk and self-management of BCRL as a factor associated with adherence; however, in addition to knowledge, adherence levels were higher in women with lower lymphedema-related distress and increased perception of self-regulation in managing distress [38]. The National Lymphedema Network, led by Deng *et al.,* conducted one of the first studies to examine sources of educational materials about lymphedema and knowledge levels of patients with primary (*n* = 517) and secondary lymphedema (*n* = 1025) [39]. Between 2006 and 2010, data were collected from participants with the completion of an online survey. Overall, participants reported that a variety of sources were used for obtaining information; however, 76% of the patients favored dedicated websites. Physician/primary health care providers were favored by 55.5%, followed by internet support groups (33.6%) and friends and family (32.1%). Participants with primary lymphedema reported lower knowledge levels about lymphedema, treatment approaches, and complications than participants with secondary lymphedema. Nurses were more often reported to be responsible for providing educational materials to participants with secondary lymphedema, with the internet being the main source for participants with primary lymphedema. Opportunities exist for health care providers to expand and utilize additional formats for providing accurate and understandable information [39]. In addition, patient-centered strategies should include education to increase awareness with attention to patients’ responses to their perceived health threat responses on both cognitive and affective levels [38].

### 3.3. Treatment Burden

The lifelong requirements of BCRL self-care are associated with patient burden, reduced quality of life (QOL), and poor adherence [2,5,6,15,16,17,18,23,26,40,41]. The components of self-management regimens can be simple to complex; however, these often culminate in significant treatment burden. Shippee *et al.* developed a framework of cumulative complexity, which defined treatment burden as an imbalance between patient “workload,” which includes day-to-day demands and responsibilities, including treatment and self-care; and patient “capacity,” which concerns the patient’s abilities to address the demands [42]. It is critical to understand the factors that create imbalance between the demands of self-care and the capacity to cope in order to prevent higher perceived treatment burden and poor patient outcomes, such as non-adherence [22].

Self-management of BCRL can only be effective if it is performed; therefore, it is necessary to better understand the complexities that directly affect women with BCRL. The components of a BCRL self-management regimen may include manual lymphatic drainage (MLD), compression garments, bandaging, skin care, and exercise [7,43]. MLD is a hands-on, light lymphatic massage that stimulates superficial lymphatic vessels to move lymph fluid from the extremity to an area where the lymphatics can drain properly [43,44]. Compression bandaging includes several layers of short stretch bandages that cover the entire limb and create an effective gradient compression to move lymph fluid out of congested areas [43]. Compression garments are personal garments that are properly fit by a trained specialist and are worn on the affected extremity to maintain or prevent progression of swelling. These garments are worn long-term. Some women with BCRL have a garment for day wear and one with a stronger compression gradient to wear during sleep as an alternative to bandaging [39]. Exercise is prescribed depending on the severity of BCRL symptoms and level of conditioning. Remedial exercises are prescribed initially when the goal is to reduce swelling in the extremity. Aerobic, strengthening, and flexibility exercises are prescribed in the self-management phase [44,45]. Skin care is essential for lymphedema management and includes meticulous hygiene and ongoing observation for breaks in the skin [39,46]. Education about risk-reduction measures to avoid exacerbation of BCRL should be included in patient teaching [8,14,44]. Low-level-laser therapy has been studied as a modality in reducing fluid volume and improving arm function in women who have BCRL; however, there are limitations to these studies in regard to sample sizes and differences in measuring objective outcomes [47,48]. The complexity of self-management regimens can be overwhelming and contribute to the everyday demands of patients with BCRL.

### 3.4. Psychosocial Impact

The examination of psychosocial adjustment and its impact on women with BCRL has been difficult to quantify due to lack of accurate measures and an operational definition [15,49]. In a systematic review, of which 19 of 23 studies were related to BCRL, Fu *et al.* examined psychosocial impact using a combination of psychological and social impact domains that directly affect an individual with lymphedema and its treatment. Operational domains for psychological impact included negative self-identity, emotional disturbance, and psychological distress. Social impact domains included marginalization by health care providers, financial burden, social isolation, perceived diminished sexuality, and public insensitivity [15]. In addition, of 11 quantitative studies, poorer social well-being was statistically significant in persons with lymphedema compared to persons without lymphedema. Of 12 qualitative studies reviewed, all described negative psychological and social impact related to lymphedema [15]. Consistent findings of a negative impact on physical and mental QOL were described by Paskett *et al.* in a literature review of articles published since 1990 (N = 726 references), of which 60 studies met inclusion criteria examining the evidence for causes, risk, prevention, diagnosis, treatment, and impact of BCRL [40]. Most studies reviewed were relevant to BCRL with conclusions as follows: (1) the need for more studies in patients with other types of cancers; (2) the need for consensus on definitions and measures; (3) increased awareness of lymphedema signs and symptoms by both patients and health care providers; and (4) the need for prompt access to care and treatment that includes psychosocial support [40]. Armer *et al.* reported specific contributors to psychological distress which included altered body image, imposed lifestyle changes and occupational role changes, and negative impact on interpersonal and family relationships [50]. Dominick *et al.* examined the impact of lymphedema-related stress on psychosocial functioning (*i.e.*, QOL and depressive symptoms) [51]. Psychosocial outcomes were measured using a data set from a cross-section of participants in the Women’s Healthy Eating and Living (WHEL) study (N = 2431 of whom 692 self-reported ever having lymphedema). Findings indicated that breast cancer survivors with lymphedema-related distress had worse physical health and mental health outcomes than women who were not distressed with lymphedema and breast cancer survivors without lymphedema [51]. The evidence of a relationship between BCRL and its negative impact on psychosocial functioning demonstrates the need for further research in developing conceptual and operational definitions, as well as measures that are more specific to psychosocial functioning.

## 4. Physiological Complexities

Poor adherence to BCRL self-care modalities is associated with a wide range of physical symptoms in which severity is measured by a grading system [52,53]. Three levels of objective criteria include: Grade I, which may present with pitting of the skin with the application of pressure and reversible edema of an extremity with elevation; Grade II, in which elevation rarely relieves edema and pitting is manifest, then later may or not demonstrate pitting due to excess fat or fibrotic skin changes; and Grade III, worsening swelling and severe thickening of the skin with the development of huge skin folds [52]. BCRL mainly affects areas of the arm, hand, breast, and trunk; however, symptoms can be present that are unable to be detected by routine clinical evaluations [41,50].

### 4.1. Symptom Burden

In addition to objective symptoms, studies have confirmed the importance of subjective symptoms, as well. Listening to patients for self-reported subjective symptoms is important in detecting BCRL at a subclinical level, if possible [45,53,54,55]. In earlier studies conducted by Armer *et al.* self-reported symptoms of heaviness and/or swelling were identified as the most common predictors of BCRL [4,27,50,56]. In a National Lymphedema Network online survey of patients with upper extremity lymphedema (*n* = 729) and lower extremity lymphedema (*n* = 1114) between March 2006 through January 2010, Ridner *et al.* reported symptoms experienced most frequently among individuals with upper extremity lymphedema were swelling (96.8%), a feeling of extremity heaviness (76.2%), current pain (67.3%), stiffness (65.8%), numbness (63.9%), and decreased range of motion (48.0%) [57]. Symptom burden is a barrier to self-management of BCRL, which is critical to prevent progression. Life-long self-management requires a plan to guide patients through survivorship with life-long support.

### 4.2. Comorbidities

There are an estimated 14 million cancer survivors among all reported cancer sites in the United States [1]. In 2015, there were an estimated 3 million women living with breast cancer in the United States [1]. Based on 2005–2011 Surveillance, Epidemiology, and End Results (SEER) program sponsored by the National Cancer Institute data, 89% of all patients diagnosed with breast cancer have lived five years or more. The majority of breast cancer survivors are 65 years of age or older [58], and although chronological age alone is not the only factor to consider when classifying older adults, it has been reported that they are three times more likely to develop BCRL than younger people [59] and are at risk for delayed diagnosis due to the coexistence of other forms of edema and comorbidities [60]. Bellury *et al.* found an interaction between symptom burden and comorbidities in 39% of older breast cancer survivors studied, [age > 70 (N = 759) and support a gero-oncology survivorship paradigm to guide care [61]. In a study of breast cancer survivors with BCRL (*n* = 74) compared with breast cancer survivors without BCRL (*n* = 75), Ridner and Dietrich reported findings that identified obesity (BMI > 30), orthopedic problems, hypertension, and arthritis as more prevalent in the lymphedema group [25]. Although pre-existing conditions present some limitations in determining causality, the findings suggested that being sedentary, compromised cardiovascular status, and the relationship of inflammatory and infectious processes with BCRL warrant further investigation [25]. In addition, co-association of medications ordered to manage comorbid conditions could also be a factor in lymphedema emergence, progression, and management [25]. The barriers to self-management of BCRL are exacerbated due to comorbidities, decreased function, lack of support, and cognitive inconsistencies and require more innovative strategies to help improve adherence to self-management of symptoms.

Through a secondary review of the qualitative studies included in this review by the first author, the most common barriers to BCRL self-management have been summarized in Table 1.

**Table 1:** Categories of factors related to decreased adherence to breast cancer-related lymphedema (BCRL) self-management.

Psychological Distress	Psychosocial Factors	Physiological Factors	Treatment Burden	Education	Comorbidities
Symptom distress	Social isolation	Heaviness of extremity	Imbalance between patient burden of treatment and their capacity to cope	Education about BCRL and self-management is not always provided	Loss of function/ROM (*i.e.*, arthritis)
Anxiety	Lack of support	Numbness	Reduced QOL	Need exists for expanding the variety of formats for BCRL education	Age
Depression	Spiritual crisis	Swelling	Decreased time for family, leisure activities due to time spent for BCRL treatment	Patient-centered strategies are needed to address both cognitive and affective levels	Cognitive changes (*i.e.*, stroke, dementia)
Emotional disturbance (*i.e.*, sadness)	Perceived diminished sexuality	Skin changes			Co-association of medications
Fear	Marginalization by health care providers	Stiffness			Sedentary lifestyle (cardiovascular implications)
Decreased perceptions of self-efficacy	Financial burden	Pain			
Stress					

## 5. Results

The following studies selected for this review are summarized in Table 2. 

**Table 2:** Summary of adherence studies.

Study	Topic/Sample	Outcome Measure	Findings
Ridner, S.H.; Bonner, C.M.; Doersam, J.K.; Rhoten, B.A.; Schultze, B.; Dietrich, M.S. Bioelectrical impedance self-measurement protocol development and daily variation between healthy volunteers and breast cancer survivors with lymphedema [62].	Home measurement program using bioelectrical impedance to establish feasibility and acceptability by patients with and without BCRL. (*n* = 11 with and *n* = 11 without BCRL)	Participant feedback used to adjust number of home measures. Participants were involved in determining feasibility of using home measures to monitor BCRL and were able to see limb volume changes.	Goal setting, informed decision-making, and experience satisfaction with outcome information relevant to limb volume measures were achieved. Ridner *et al.* suggests patients’ perception of a lack of results in self-care and subsequent feelings of decreased self-efficacy lead to poor adherence.
Armer, J.; Shook, R.P.; Schneider, M.K.; Brooks, C.W.; Peterson, J.; Stewart, B.R. Enhancing supportive-educative nursing systems to reduce risk of post-breast cancer lymphedema [27].	Prospective surveillance study to assess for BCRL with self-care using manual lymphatic drainage (MLD). (N = 27)	Motivational interviewing and solution-focused therapy.	When participants were found to be non-adherent to the MLD intervention, motivational interviewing and solution-focused therapy enabled staff to identify strengths and weaknesses associated with non-adherence.
Brown, J.; Cheville, A.; Tchou, J.C.; Harris, S.R.; Schmitz, K.H. Prescription and adherence to lymphedema self-care modalities among women with breast cancer-related lymphedema [9].	Adherence to BCRL self-care modalities at 3-, 6-, and 12-month intervals. (N = 141)	A questionnaire developed to assess adherence to self-care modalities. Adherence = percentage of time that self-care modalities were completed at the frequency recommended by the lymphedema therapist. Adherence ≥ 75%.	At 12 months, adherence was sub-optimal at 69%. Results identified a need for an infrastructure of support and education.
Forner-Cordero, I.; Muñoz-Langa, J.; Forner-Cordero, A.; DeMiguel-Jimeno J. Predictive factors of response to decongestive therapy in patients with breast-cancer-related lymphedema [63].	Adherence to bandaging during combined decongestive therapy (CDT). (N = 171)	Bandaging of the extremity at home and arriving for therapy each day with bandages in place constituted adherence. Adherence was assigned percentages as follows: 90% = Good 60%–89% = Fair >60% = Bad	Adherence to bandaging during CDT was predictive of better treatment outcomes.
Tidhar, D.; Katz-Leurer M. Aqua lymphatic therapy in women who suffer from breast cancer treatment-related lymphedema: a randomized controlled study [64].	Comparison of adherence, limb volume, and QOL in women who perform only self-management treatment for BCRL and those who perform self-management treatment for BCRL and aqua lymphatic therapy (ALT). (*n* = 16 study group; *n* = 32 control group).	Adherence diary based on attendance based on an assumption of 50% adherence in the control group and 85% in the ALT group. Limb volume measures and QOL questionnaires were also used.	The mean adherence rate to self-management for both groups was lower than 30% at entry time and during the study period. The adherence for ALT was 79%. Eighty-six percent of the women adhered to more than 75% of the ALT sessions. This was significantly higher compared with self-management therapy and each of its components (*p* < 0.05).
Letellier, M.E.; Towers, A.; Shimony, A.; Tidhar D. Breast cancer-related lymphedema: A randomized controlled pilot and feasibility study [65].	Comparison of home-based exercise to home-based exercise and weekly aqua lymphatic therapy (ALT). (ALT group *n* = 13; control group *n* = 12)	Diaries used to measure adherence. Arm disability, pain intensity scores, and QOL were also examined.	The ALT group demonstrated a significant difference over the home exercise alone group (control) with a reduction in pain intensity scores, arm disability, and increased QOL. Association of adherence with self-management practices and outcome measures were prohibited due to a 52% return rate of diaries.
Sherman, K.; Koelmeyer, L. The Role of Information Sources and Objective Risk Status on Lymphedema Risk-Minimization Behaviors in Women Recently Diagnosed With Breast Cancer [31].	A measure of demographics, lymphedema knowledge, lymphedema information sources used, and adherence to risk-minimization recommendations in women recently diagnosed with breast cancer. (N = 106)	A survey questionnaire of 12 self-report items was administered at the time of surgery and 3-months post-operatively. For each recommendation practice, a score of 1 was given, with a total score summed out of 12.	Women breast cancer survivors at risk for BCRL scored high on performing most BCRL risk-reduction activities. Mean total adherence was 9.53, with 32 women performing every recommendation and 2 performing none. The scale demonstrated a high internal consistency with a Cronbach alpha of 0.86.
Sherman, K.; Miller, S.; Roussi, P.; Taylor, A. Factors predicting adherence to risk management behavior of women at increased risk for developing lymphedema [38].	Adherence to risk minimization behaviors and psycho-educational factors was assessed. (N = 103)	Adherence was measured using a 12-item self-report yes/no dichotomous items based on the ACS lymphedema risk management guidelines. The survey questionnaire was administered at baseline, 6-, and 12-months after giving printed information about breast cancer.	Women breast cancer survivors who understand BCRL risk and feel confident in managing it are more likely to adhere to recommended strategies. The study demonstrated an increase in knowledge over time, lower distress, and higher self-efficacy and self-regulation abilities.

Ridner *et al.* suggested BCRL self-management adherence rates of less than 50% from previous study participants. They identified patients’ perception of a lack of results from self-care (*i.e.*, arm volume measurements) and feelings of decreased self-efficacy as reasons for poor self-care adherence [62]. In a recent study, a home measurement program using bioelectrical impedance was piloted (*n* = 11 women with BCRL and *n* = 11 women without BCRL). It was theorized that a home measurement system would provide the ability to set self-care goals, reinforce care with measureable results, allow informed decision-making, and experience satisfaction with outcome information [62]. Although compliance rates were not calculated, an adjustment in the number of times for self-measurement was made based on participant feedback. Overall, feasibility of the home measurement system was demonstrated and it was accepted by the participants and captured limb volume change. It may be able to lend support with monitoring BCRL treatment and clinical trials are warranted [62].

Armer *et al.* conducted a prospective surveillance study in which 27 participants were enrolled. The participants were assessed for symptoms of BCRL pre- and post-operatively and every six months for 18 months. Based on feedback from the parent study, which indicated that patients were not performing the self-care task of manual lymphatic drainage as they had been instructed, it became clear that an enhancement to the current intervention was needed. Motivational interviewing and solution-focused therapy were interactive activities that were implemented by the study nurses. Through interactive strategies, non-compliance was addressed, allowing for rapport building between the patients and the nurses, a clinical assessment, and discussion to summarize and identify advantages and disadvantages to care. Solution-focused therapy was achieved through dialogue with the nurses who empowered and motivated patients to become engaged in the self-care goal-setting process. The addition of motivational interviewing and solution-focused therapy enabled the study staff to identify strengths and weaknesses in the participants’ abilities to develop self-care agency or the power to engage in self-care [27].

A third study conducted by Brown *et al.* specifically profiled prescription and adherence with 141 breast cancer survivors with BCRL who had been in a previous physical and activity trial (PAL) using a 12-month randomized weightlifting trial [15]. A questionnaire developed to assess adherence to self-care modalities was administered at baseline, three-, six-, and 12-month intervals. Adherence was defined as the percentage of time that self-care modalities were completed at the frequency recommended by the lymphedema therapist. At 12 months, overall adherence to all self-care modalities was not optimal with the majority of the participants (69%) reporting an adherence rate of less than 75%. The study concluded there was a need for an infrastructure for BCRL education and support, such as that which exists for patients with Type I diabetes [9].

Forner-Cordero *et al.* studied adherence to bandaging during combined decongestive therapy (CDT) with 171 patients with BCRL. The endpoint of the study was the percentage of limb volume reduction at the end of the CDT period [63]. Adherence to bandaging was acknowledged daily by the physician. Bandages were removed each session and adherence to reapplying the bandages at home per protocol and arriving the next day with the bandages in place constituted adherence. Percentages were assigned as a measure of adherence; “good” adherence was when the patient maintained the bandages 90% of the time of treatment, “fair” between 60% and 89%, and “bad” with less than 60% of the time. Adherence to bandaging during CDT was predictive of better limb volume reduction [63].

Tidar and Katz-Leurer used treatment diaries as a measure of adherence with an aqua lymphatic therapy (ALT) intervention. ALT was the intervention in a study to examine whether there were differences in adherence, limb volume, and QOL between women who performed only self-management treatment for BCRL and women who performed self-management for BCRL and ALT [64]. ALT is a method that uses the viscosity of water to provide resistance to body movement. Hydrostatic pressure is used to protect the arm from swelling and reduces edema. Groups of patients with BCRL attended 45-min sessions in a pool and performed breathing and self-massage techniques in the water in sequence. An immediate mean arm volume reduction of 16% (53 mL) of the affected arm was reported after the first ALT session, and a reduction of 29% (98.2 mL) after the last ALT session (*n* = 16 ALT; *n* = 32 control). Adherence to therapy was the main outcome in this study; therefore, calculation of an adequate sample size was based on an the assumption of an approximate 50% adherence rate in the control group, as compared to 85% in the study group (based on earlier studies by Boris and Lasinski) [64]. Additionally, Letellier, Shimony, and Tidhar conducted a second study with ALT to compare weekly ALT and home-based exercise to home-based exercise alone (*n* = 13 ALT and *n* = 12 control) for 12 weeks. Home-based exercises were performed using an instructional DVD. Diaries were used to measure adherence with a 52% return rate (*n* = 13 from both groups), which prohibited the ability to look at an association of adherence with self-management practices and outcome measures; however, ALT demonstrated a significant difference over home-based exercise alone with a reduction in pain intensity scores, arm disability and an increase in QOL [65].

Studies that are conducted to understand factors that influence and lead to initiation and maintenance of self-management strategies are limited; however, psychological and psychosocial factors have begun to emerge as indicators of adherence [15,16,18,31,38,51]. In addition, there are a growing number of studies in the area of patient knowledge of BCRL self-care practices and the impact on adherence. Sherman and Koelmeyer conducted a study of 106 women diagnosed with breast cancer and at risk for BCRL in an effort to assess the role of educational resources and objective risk status on knowledge and BCRL risk-minimization behaviors [31]. A survey questionnaire was administered at the time of surgery and three-months post-operatively to measure demographics, lymphedema knowledge, lymphedema information sources used, and adherence to risk-minimization recommendations. Adherence to risk-minimization behaviors was addressed with 12 self-report items which were based on national recommendation guidelines for lymphedema. For each recommendation practice, a score of 1 (yes)/0 (no) was given, with a total score summed out of 12 possible. High internal consistency with this scale was demonstrated with a Cronbach alpha of 0.86. The mean total adherence was 9.53 (SD = 2.95; range 0–12), with 32 women performing every recommendation and two performing none. The highest level of non-adherence, reported in 28% of the participants, was in seeking medical assistance with the emergence of BCRL symptoms, wearing gloves for housework or gardening, and using an electric razor when shaving the axillae. Other outcomes measures demonstrated that knowledge was high and increased over time and receiving information from nursing staff three months post-operatively was significant in predicting risk-minimization behaviors [31].

Sherman *et al.* have recently published a study (*N* = 103) which expands on their previous research and investigates psycho-educational factors associated with BCRL risk [38]. Adherence to BCRL risk-minimization behaviors is again assessed using a 12-item self-report scale. Psycho-educational factors were measured at baseline and then again at six- and 12-months after giving participants an American Cancer Society publication entitled, “Lymphedema: What Every Woman with Breast Cancer Should Know.” Findings demonstrated an increase in knowledge over time, lower distress, and higher self-efficacy and self-regulatory abilities to manage stress; all were associated with increased adherence [38].

## 6. Discussion

There are few studies in the literature that utilize a valid and reliable measure of patient adherence to self-management of BCRL. Adherence is used in the medical sense as a definition of success in the form of a treatment response or physical change, most often referring to limb volume. A positive treatment response of a 50% decrease in limb volume assumes that a patient has chosen to adhere to the study activities. This may or may not be the case and it is necessary to differentiate between physical responses and behavioral responses, especially when dealing with chronic conditions requiring life-long maintenance. Several studies from the behavioral sciences indicate that behavioral self-monitoring, a method of self-observation, evaluation, and recording of one’s behavior, is used by 80% of cognitive and behavioral therapists to help people to make behavioral changes [66]. Similar to Leventhal’s Common Sense Model, adherence to self-management of BCRL requires women to perform self-observation, adopt risk-reduction and symptom management activities on a regular basis, and, in some fashion, record their behavior for later reappraisal of outcomes [33]. There are many theoretical frameworks that can be applied to behavior change relevant to self-care; however, there is a lack of reliable and valid measures that can be used to evaluate the concept of adherence.

## 7. Conclusions

BCRL research has grown significantly in understanding the barriers and facilitators to self-management. Recent studies have reported that psychological and psychosocial factors are contributors to poor adherence to BCRL risk-minimization behaviors and treatment regimens. Further research is needed to advance the body of knowledge in the area of instrument development to measure outcomes to behavioral change relevant to adherence and successful management of chronic diseases and conditions, such as BCRL. Health care providers have a responsibility to provide resources to help patients learn about their health and how best to manage it. An understanding of patient care on a multi-dimensional level is necessary to build rapport and anticipate and provide adequate resources. Patient engagement in taking an active role in understanding their health and plan of care may help to increase BCRL adherence with meaningful measures of successful outcomes.

## References

[1] (2015). Cancer Treatment & Survivorship Facts & Figures 2014–2015. http://www.cancer.org/acs/groups/content/@research/documents/document/acspc-042801.pdf.

[2] Armer J.M., Henggeler M.H., Brooks C.W., Zagar E.A., Homan S., Stewart B.R. (2008). The health deviation of post-breast cancer lymphedema: Symptom assessment and impact on self-care agency. Self Care Depend. Care Nurs..

[3] Khan S. (2009). Axillary reverse mapping to prevent lymphedema after breast cancer surgery: Defining the limits of the concept. J. Clin. Oncol..

[4] Armer J.M., Radina M.E., Porock D., Culbertson S.D. (2003). Predicting breast cancer-related lymphedema using self-reported symptoms. Nurs. Res..

[5] Fu M.R., Chen C.M., Haber J., Guth A.A., Axelrod D. (2010). The effect of providing information about lymphedema on the cognitive and symptom outcomes of breast cancer survivors. Ann. Surg. Oncol..

[6] Ridner S.H., Fu M.R., Wanchai A., Stewart B.R., Armer J.M., Cormier J.N. (2012). Self-management of lymphedema: A systematic review of the literature from 2004 to 2011. Nurs. Res..

[7] Lasinski B.B., McKillip Thrift K., Squire D., Austin M.K., Smith K.M., Wanchai A., Green J.M., Stewart B.R., Cormier J.N., Armer J.M. (2012). A systematic review of the evidence for complete decongestive therapy in the treatment of lymphedema from 2004 to 2011. Am. J. Phys. Med. Rehab..

[8] Armer J.M., Brooks C.W., Stewart B.R. (2011). Limitations of self-care in reducing the risk of lymphedema: Supportive-educative systems. Nurs. Sci. Quart..

[9] Brown J.C., Cheville A.L., Tchou J.C., Harris S.R., Schmitz K.H. (2014). Prescription and adherence to lymphedema self-care modalities among women with breast cancer-related lymphedema. Support Care Cancer.

[10] Haynes R., Sackett D. (1976). Compliance with Therapeutic Regimens.

[11] Lutfey K., Wishner W. (1999). Beyond “compliance” is “adherence”: Improving the prospect of diabetes care. Diabetes Care.

[12] Robinson J.H., Callister L.C., Berry J.A., Dearing K.A. (2008). Patient-centered care and adherence: Definitions and applications to improve outcomes. J. Am. Acad. Nurse Pract..

[13] Vermeire E., Hearnshaw H., van Royen P., Denekens J. (2001). Patient adherence to treatment: Three decades of research. A comprehensive review. J. Clin. Pharm. Ther..

[14] Armer J.M., Hulett J.M., Bernas M., Ostby P., Stewart B.R., Cormier J.N. (2013). Best-practice guidelines in assessment, risk reduction, management, and surveillance for post-breast cancer lymphedema. Curr. Breast Cancer Rep..

[15] Fu M.R., Ridner S.H., Hu S.H., Stewart B.R., Cormier J.N., Armer J.M. (2013). Psychosocial impact of lymphedema: A systematic review of literature from 2004 to 2011. Psychooncology.

[16] Fu M.R., Kang Y. (2013). Psychosocial impact of living with cancer-related lymphedema. Semin. Oncol. Nurs..

[17] Ridner S.H., Dietrich M.S., Kidd N. (2011). Breast cancer treatment-related lymphedema self-care: Education, practices, symptoms, and quality of life. Support Care Cancer.

[18] Sherman K.A., Koelmeyer L. (2013). Psychosocial predictors of adherence to lymphedema risk minimization guidelines among women with breast cancer. Psychooncology.

[19] Boris M., Lasinski B. (1994). Lymphedema reduction by noninvasive complex lymphedema therapy. Oncology.

[20] Stout N.L., Pfalzer L.A., Springer B., Levy E., McGarvey C.L., Danoff J.V., Gerber L.H., Soballe P.W. (2012). Breast cancer-related lymphedema: Comparing direct costs of a prospective surveillance model and a traditional model of care. Phys. Ther..

[21] Norman D. (2010). Living with Complexity.

[22] Ridgeway J.L., Egginton J.S., Tiedje K., Linzer M., Boehm D., Poplau S., de Oliveira D.R., Odell L., Montori V.M., Eton D.T. (2014). Factors that lessen the burden of treatment in complex patients with chronic conditions: A qualitative study. Patient Prefer Adher..

[23] Fu M.R., Rosedale M. (2009). Breast cancer survivors' experiences of lymphedema-related symptoms. J. Pain Symptom Manag..

[24] National Comprehensive Cancer Network (NCCN) (2015). Distress management guidelines. http://www.nccn.org/professionals/physician_gls/pdf/distress.pdf.

[25] Ridner S.H., Dietrich M.S. (2008). Self-reported comorbid conditions and medication usage in breast cancer survivors with and without lymphedema. Oncol. Nurs. Forum.

[26] Rosedale M., Fu M.R. (2010). Confronting the unexpected: Temporal, situational, and attributive dimensions of distressing symptom experience for breast cancer survivors. Oncol. Nurs. Forum.

[27] Armer J.M., Shook R.P., Schneider M.K., Brooks C.W., Peterson J., Stewart B.R. (2009). Enhancing Supportive-Educative Nursing Systems to Reduce Risk of Post-Breast Cancer Lymphedema. Self Care Depend. Care Nurs..

[28] McWayne J., Heiney S.P. (2005). Psychologic and social sequelae of secondary lymphedema. Cancer.

[29] Paskett E.D., Stark N. (2000). Lymphedema: Knowledge, Treatment, and Impact Among Breast Cancer Survivors. Breast J..

[30] Ridner S.H. (2005). Quality of life and a symptom cluster associated with breast cancer treatment-related lymphedema. Support Care Cancer.

[31] Sherman K., Koelmeyer L. (2011). The role of information sources and objective risk status on lymphedema risk-minimization behaviors in women recently diagnosed with breast cancer. Oncol. Nurs. Forum.

[32] Tsuchiya M., Horn S., Ingham R. (2012). Information provision and problem-solving processes in Japanese breast cancer survivors with lymphoedema symptoms. Scand. J. Caring Sci..

[33] Leventhal H., Meyer D., Nerenz D., Rachman S. (1980). The common sense representation of illness danger. Contributions to Medical Psychology.

[34] Leventhal H., Safer M.A., Cleary P.D., Gutmann M. (1980). Cardiovascular risk modification by community-based programs for life-style change: Comments on the Stanford study. J. Consult Clin. Psychol..

[35] Lau R.R., Hartman K.A. (1983). Common sense representations of common illnesses. Health Psychol..

[36] Leventhal H., Leventhal E., Cameron L., Baum A., Revenson T., Singer J. (2001). Representations, procedures, andeffect in illness self-regulation a perceptual cognitive model. Handbook of Health Psychology.

[37] Leventhal H., Leventhal E.A., Breland J.Y. (2011). Cognitive Science Speaks to the “Common-Sense” of Chronic Illness Management. Ann. Behav. Med..

[38] Sherman K.A., Miller S.M., Roussi P., Taylor A. (2015). Factors predicting adherence to risk management behaviors of women at increased risk for developing lymphedema. Support Care Cancer.

[39] Deng J., Fu M.R., Armer J.M., Cormier J.N., Radina E., Thiadens S.R., Dietrich M.S., Weiss J., Tuppo C.M., Ridner S.H. (2013). Self-reported information sources and perceived knowledge in individuals with lymphedema. Lymphology.

[40] Paskett E.D., Dean J.A., Oliveri J.M., Harrop J.P. (2012). Cancer-related lymphedema risk factors, diagnosis, treatment, and impact: A review. J. Clin. Oncol..

[41] Cormier J., Xing Y., Zaniletti I., Askew R., Stewart B., Armer J. (2009). Minimal limb volume change has a significant impact on breast cancer survivors. Lympihology.

[42] Shippee N.D., Shah N.D., May C.R., Mair F.S., Montori V.M. (2012). Cumulative complexity: A functional, patient-centered model of patient complexity can improve research and practice. J. Clin. Epidemiol..

[43] Lasinski B.B. (2013). Complete decongestive therapy for treatment of lymphedema. Semin. Oncol. Nurs..

[44] National Lymphedema Network (2015). The diagnosis and treatment of lymphedema. http://www.lymphnet.org/lymphedemaFAQs/positionPapers.htm.

[45] Stout Gergich N.L., Pfalzer L.A., McGarvey C., Springer B., Gerber L.H., Soballe P. (2008). Preoperative assessment enables the early diagnosis and successful treatment of lymphedema. Cancer.

[46] National Cancer Institute (2015). Lymphedema PDQ. http://www.cancer.gov/cancertopics/pdq/supportivecare/lymphedema/healthprofessional.

[47] Kaviani A., Fateh M., Yousefi Nooraie R., Alinagi-zadeh M.R., Ataie-Fashtami L. (2006). Low-level laser therapy in management of postmastectomy lymphedema. Lasers Med. Sci..

[48] Omar M., Shaheen A., Zafar H. (2012). A systematic review of the effect of low-level laser therapy in the management of breast cancer-related lymphedema. Support Care Cancer.

[49] Heppner P., Tierney C., Armer J., Whitlow N., Reynolds A. (2009). Breast cancer survivors coping with lymphedema: What all counselors need to know. J. Couns. Dev..

[50] Armer J., Fu M.R., Wainstock J.M., Zagar E., Jacobs L.K. (2004). Lymphedema following breast cancer treatment, including sentinel lymph node biopsy. Lymphology.

[51] Dominick S.A., Natarajan L., Pierce J.P., Madanat H., Madlensky L. (2014). The psychosocial impact of lymphedema-related distress among breast cancer survivors in the WHEL Study. Psychooncology.

[52] International Society of Lymphology (2013). The diagnosis and treatment of peripheral lymphedema: 2013 Consensus Document of the International Society of Lymphology. Lymphology.

[53] Ostby P.L., Armer J.M., Dale P.S., van Loo M.J., Wilbanks C.L., Stewart B.R. (2014). Surveillance recommendations in reducing risk of and optimally managing breast cancer-related lymphedema. J. Pers. Med..

[54] Armer J.M., Stewart B.R. (2010). Post-breast cancer lymphedema: Incidence increases from 12 to 30 to 60 months. Lymphology.

[55] Hulett J., Armer J., Stewart B., Wanchai A. (2015). Perspectives of the Breast Cancer Survivorship Continuum: Diagnosis through 30 Months Post-Treatment. J. Pers. Med..

[56] Armer J.M., Whitman M. (2002). The problem of lymphedema following breast cancer treatment: Prevalence, symptoms, & self-management. Lymphology.

[57] Ridner S.H., Deng J., Fu M.R., Radina E., Thiadens S.R., Weiss J., Dietrich M.S., Cormier J.N., Tuppo C.M., Armer J.M. (2012). Symptom burden and infection occurrence among individuals with extremity lymphedema. Lymphology.

[58] National Cancer Institute (2015). Surveillance, Epidemiology, and End Results Program. http://seer.cancer.gov.

[59] Nazarko L. (2006). Understanding lymphoedema in older people. Nurs. Resid. Care.

[60] Armer J.M., Stewart B.R., Wanchai A., Lasinski B.B., Smith K.M., Cormier J.N. (2012). Rehabilitation Concepts Among Aging Survivors Living With and At Risk for Lymphedema. Top. Geriat Rehabil..

[61] Bellury L., Ellington L., Beck S.L., Pett M.A., Clark J., Stein K. (2013). Older breast cancer survivors: Can interaction analyses identify vulnerable subgroups? A report from the American Cancer Society Studies of Cancer Survivors. Oncol. Nurs. Forum.

[62] Ridner S.H., Bonner C.M., Doersam J.K., Rhoten B.A., Schultze B., Dietrich M.S. (2014). Bioelectrical impedance self-measurement protocol development and daily variation between healthy volunteers and breast cancer survivors with lymphedema. Lymphat. Res. Biol..

[63] Forner-Cordero I., Muñoz-Langa J., Forner-Cordero A., DeMiguel-Jimeno J. (2010). Predictive factors of response to decongestive therapy in patients with breast-cancer-related lymphedema. Ann. Surg. Oncol..

[64] Tidhar D., Katz-Leurer M. (2010). Aqua lymphatic therapy in women who suffer from breast cancer treatment-related lymphedema: A randomized controlled study. Support Care Cancer.

[65] Letellier M.E., Towers A., Shimony A., Tidhar D. (2014). Breast cancer-related lymphedema: A randomized controlled pilot and feasibility study. Am. J. Phys. Med. Rehabil..

[66] Olson R., Schmidt S., Winkler C., Wipfli B. (2011). The effects of target behavior choice and self-management skills training on compliance with behavioral self-monitoring. Am. J. Health Promot..

